# Strategies for Targeting Gene Therapy in Cancer Cells With Tumor-Specific Promoters

**DOI:** 10.3389/fonc.2020.605380

**Published:** 2020-12-14

**Authors:** Mariela Montaño-Samaniego, Diana M. Bravo-Estupiñan, Oscar Méndez-Guerrero, Ernesto Alarcón-Hernández, Miguel Ibáñez-Hernández

**Affiliations:** ^1^ Laboratorio de Terapia Génica, Departamento de Bioquímica, Escuela Nacional de Ciencias Biológicas del Instituto Politécnico Nacional, Ciudad de México, México; ^2^ Laboratorio de Genética Molecular, Departamento de Bioquímica, Escuela Nacional de Ciencias Biológicas del Instituto Politécnico Nacional, Ciudad de México, México

**Keywords:** cancer, gene therapy, targeted treatment, specific promoters, non-viral vectors

## Abstract

Cancer is the second cause of death worldwide, surpassed only by cardiovascular diseases, due to the lack of early diagnosis, and high relapse rate after conventional therapies. Chemotherapy inhibits the rapid growth of cancer cells, but it also affects normal cells with fast proliferation rate. Therefore, it is imperative to develop other safe and more effective treatment strategies, such as gene therapy, in order to significantly improve the survival rate and life expectancy of patients with cancer. The aim of gene therapy is to transfect a therapeutic gene into the host cells to express itself and cause a beneficial biological effect. However, the efficacy of the proposed strategies has been insufficient for delivering the full potential of gene therapy in the clinic. The type of delivery vehicle (viral or non viral) chosen depends on the desired specificity of the gene therapy. The first gene therapy trials were performed with therapeutic genes driven by viral promoters such as the CMV promoter, which induces non-specific toxicity in normal cells and tissues, in addition to cancer cells. The use of tumor-specific promoters over-expressed in the tumor, induces specific expression of therapeutic genes in a given tumor, increasing their localized activity. Several cancer- and/or tumor-specific promoters systems have been developed to target cancer cells. This review aims to provide up-to-date information concerning targeting gene therapy with cancer- and/or tumor-specific promoters including cancer suppressor genes, suicide genes, anti-tumor angiogenesis, gene silencing, and gene-editing technology, as well as the type of delivery vehicle employed. Gene therapy can be used to complement traditional therapies to provide more effective treatments.

## Introduction

### Cancer Basics and Available Treatments. Why Use Gene Therapy?

Nowadays, cancer is one of the main causes of death worldwide. According to the World Health Organization (WHO), cardiovascular disease related deaths are the main cause of death worldwide, being cancer in the second place, responsible for one in six deaths. Nevertheless, it is believed that in the future it could become the first cause of death (1, 2). Cancer is not exclusive of high economy level countries; developing countries contribute with a little more than a half (56%) of new cancer diagnosis per year and with 64% of deaths due to cancer worldwide. Hence, it is considered as an important obstacle to the economic and social development among all countries. WHO estimates that by the year 2030 cancer cases could surpass 20 million cases per year worldwide, as a consequence of the current demographic exPLoSion and the increase of elderly people. Nonetheless, there is a possibility of diminishing these figures given that more than 30% of all types of cancer are preventable by avoiding the main risk factors such as smoking, alcoholism, unhealthy diets, and sedentary lifestyle plus some chronic infectious diseases, especially those of viral nature (2).

Vaccination and early diagnosis along with proper therapies are important aspects to be taken into consideration to significantly reduce cancer related deaths. All the same, these strategies have not been successful given tumor variability and complexity. Regardless of the neoplasms origins, the main features common to all tumor cells include: continuous proliferative signaling, tumor suppressors evasion, apoptosis resistance, replicative immortality, cell-energetics deregulation, metastasis, and angiogenesis activation and immune system evasion (3). Surgery and radiotherapy are the treatments used to treat local non-metastatic tumors whereas antineoplastic medicine such as chemotherapy, hormones, and biological therapies are preferably used to handle metastatic tumors. Toxicity rendered by chemotherapeutic drugs, which induce undesirable massive destruction of normal cells, next to the upgrowing knowledge of molecular biology of tumor cells and the exclusive tumor features, have arisen the need of searching alternative targeted and efficient treatments against cancer, being gene therapy one of the most promising procedures for accomplishing such a purpose. Gene therapy consists in the introduction of therapeutic nucleic acids (TNAs) into target cells in order to achieve a beneficial molecular effect for patients. TNAs’ delivery into cancer cells, such as genes, oligonucleotides, or interference RNAs, has enabled cancer battling by means of gene substitution, or genetic expression regulation either over-expression or repression (4–8). Notwithstanding, TNAs effect is transient and most of the time the desired effect has not been achieved. Because of this, expression plasmid vectors have been designed and constructed with the purpose of avoiding transient effects of TNAs. At first, therapeutic genes were under transcriptional control of ubiquitous eukaryotic viral promoters such as those from cytomegalovirus (CMV), Rous sarcoma virus (long terminal repeat, LTR), simian virus 40 (SV-40), and Epstein–Barr virus (EB) (9), which mediate non-specific expression of therapeutic genes in neoplastic cells and normal cells likewise. For this reason, the need of designing new expression systems with cancer/tissue specific promoters in order to drive gene expression of therapeutic genes towards target cells has arisen.

Cancer/tumor-specific promoters have been used to perform gene therapy in many types of neoplasia; within the most studied ones we have hepatocellular carcinoma, breast, lung, colorectal, pancreas and prostate cancer (8, 10–13). Gene therapy success in cancer treatment relies not only on a good molecular strategy, which consists of the design of specific genetic material being exclusively expressed within tumor cells, but also on the need of a safe, efficient and specific gene delivery system. Accordingly, a wide variety of genetic vectors have been developed for TNAs delivery, within them, viral vectors have shown the highest efficiencies but their greatest disadvantage is their immunogenicity. In this sense, non-viral vectors have proven to be safer when it comes to *in vivo* TNAs delivery, even when they are less efficient. So, the search of a safer, more efficient and specific genetic vector is still going on.

The aim of the present review is to provide a general view of the most recent molecular strategies in which cancer/tumor-specific promoters have been used in the design and construction of appropriate genetic vectors so targeted gene therapy can be performed on specific neoplasia.

## Current Molecular Strategies in Cancer Gene Therapy

Gene transferring technologies allow a wide variety of treatment possibilities which can be used in complementing conventional therapies as well as providing new treatment strategies. New delivery systems as well as more sophisticated gene expression systems are being studied with the aim of achieving cancer treatment and removal. That is why the use of nucleic acids such as recombinant DNA, interfering RNA (iRNA), microRNAs (miRNAs), zinc finger nucleases (ZFNs), transcription activator-like effector nucleases (TALENs), clustered regularly interspaced short palindromic repeats/CRIPR-associated protein 9 (CRISPR/Cas), and suicide genes have aroused great interest among the scientific community (14–16).

### Antisense Oligonucleotides Technology in Cancer

Antisense oligonucleotides (ASOs) are defined as single-stranded, highly modified, synthetic RNA or DNA oligonucleotides designed to selectively bind to target RNA molecules, which are encoded by the gene of interest, through Watson–Crick base-pairing with the purpose of modulating its function (17, 18). Binding of ASO to its complementary target can trigger different action mechanisms (16). These mechanisms can be classified as those that bind to RNA and interfere with its function without promoting RNA degradation and those that promote RNA degradation (19, 20). Even when several ASO-based treatment candidates have gone into different clinical trial stages, none has been approved for cancer treatments yet (18). Despite this, ASO-mediated intervention is a potential therapeutic approach for targeted manipulation of gene expression for cancer treatment.

### Interfering RNAs 

RNA interference technology has made considerable progress, especially when it comes to cancer treatment (21), since first described in *Caenorhabditis elegans* in 1998 (22). RNAi is a double-stranded RNA (dsRNA)-based gene silencing technology that evolved as a natural cell defense mechanism against RNA viruses. This mechanism identifies pathogenic dsRNA molecules and targets them for cleavage. Up to now, three classes of small RNAs have been described in animals: microRNAs (miRNAs), small interfering RNAs (siRNAs), and PIWI-interacting RNAs (piRNAs) (23). Usually, these RNAs guide Argonaute proteins to target RNAs *via* Watson–Crick base-pairing, usually resulting in gene silencing (24).

The miRNAs are single-stranded, non-coding RNA molecules, of 21–22 nucleotides in length, which main function is silencing gene expression at a post-transcriptional level by the imperfect binding to the target mRNA, specifically in nucleotides 2–8 of the miRNA, known as the seed region (25, 26). In cancer, some miRNAs are over-expressed, inducing tumor development (oncomirs) and others are downregulated, blocking inhibitory control over some oncogenes, or cell differentiation, and apoptosis control (tumor suppressor miRNAs) (27–29).

The siRNAs are synthetic double-stranded RNA molecules of 21–23 nucleotides in length which induce gene silencing at post-transcriptional levels by binding to the target mRNA in specific binding sites which leads it to its degradation and thus to translation inhibition (30–33). It is worth mentioning that siRNA union with its target mRNA is highly selective and when compared against miRNAs, this union is 100% complementary, discriminating sequences even with one different nucleotide (34).

The piRNAs are a kind of small non-coding RNA of 24–32 nucleotides in length, named this way because of their interactions with the PIWI subfamily of Argonaut proteins which exerts a transposon gene silencing effect besides other kinds of regulation such as epigenetics, gene and protein regulation, genome rearrangement, spermatogenesis, and germ stem cells maintenance (35–37). The piRNAs can be mainly involved in epigenetic regulation rather than post-transcriptional regulation of many biological phenomena such as cancer (38, 39).

RNA interference therapies also include small hairpin RNAs (shRNAs) (40), which are RNA molecules that can be synthesized from expression vectors within cell nucleus, to be then transported to the cytoplasm and processed by endogenous machinery to give siRNAs. Each shRNA can encode more than two siRNAs to silence the target mRNA (41, 42). The shRNAs can be transcribed by type II or type III RNA polymerase throughout type II RNA polymerase depending promoters or type III RNA polymerase depending promoters in the expression cassette design to carry on RNA interference (40, 41).

### Gene Editing Techniques in Cancer 

Development of gene editing techniques has enabled the possibility of directly targeting and modifying specific gene sequences in almost every eukaryotic cell, displaying an enormous potential for its use in many fields which range from basic research to applied biomedicine and biotechnology (43). The latest advances in the development of programmed nucleases such as ZFNs, TALENs, and CRISPR/Cas have enormously accelerated transition from basic research to the advances in gene edition within clinical practice (15).

ZFNs are proteins which arise from the specific union of a Cys_2_-His_2_ protein and the Fok1 restriction endonuclease cleavage domain resulting in DNA target sequence cleavage (44–46). On the other hand, there are transcription activator-like effector nucleases (TALENs); these ones arise from the binding of a DNA binding domain and a nuclease catalytic domain of Fok1 (47). Using gene editing techniques such as ZFNs and specific TALENs, has been specifically inhibiting cervix cancer cell growth (48–50) and acute lymphoblastic leukemia (45, 51). As well, ZFNs have been used for fighting the resistance to therapeutic agents in breast cancer cells (52).

The most recent gene editing technology developed is CRISPR/Cas. It is originally present in bacteria and archaea as an “immune system” to protect these organisms against phage and other viral infections. One interesting feature of CRISPR systems is that they use a guide RNA (gRNA) that binds to the DNA target site while a nuclease known as CRISPR-associated caspase protein (Cas) cleaves specific DNA strands which are complementary to the RNAg of the CRISPR system (53). This gene editing system can be used for raising adaptive immunity, fighting carcinomas and specific mutation editing (54). It has been shown that using this gene editing technique, the reduction of tumor size, migration capacity, and drug resistance can be achieved in pancreatic (55), prostate (56–58), colon (59), and breast cancers (60, 61).

The potential of gene editing techniques for its use in basic research as well as in clinical cancer treatment has begun to develop strongly. In the future, grouped gRNA will provide a complete set of susceptible genes which could be modified in most cancer cell lines (62). This resource, along with the available information about cancer cell line genetics and epigenetics will allow us to achieve new safer, more efficient cancer gene therapies.

### Suicide Gene Therapy 

Suicide gene therapy is based on introducing suicide genes to express enzymes or proteins that trigger the death of tumor cells (63–65), directly or indirectly. Direct suicide gene therapy consists of a gene that encodes for a protein that is cytotoxic and when expressed within the tumor cell induces its death. This has been achieved with the diphtheria toxin A complete gene or with segments of it, whose expression can change cell membrane stability and reduce tumor cell viability (14, 66–68). Indirect suicide gene therapy uses genes that express enzymes such as the Herpes Simplex Virus Thymidine Kinase (HSV-tk) accompanied by the administration of ganciclovir (GCV) (65, 69, 70). Another gene that has been used is the *Escherichia coli* cytosine deaminase gene; it encodes for an enzyme that catalyzes conversion of 5-fluorocytosine (5-FC) into 5-fluoruracile (5-FU), a drug used in conventional chemotherapy of HCC, prostate, colon, and breast cancers because it causes cell proliferation inhibition and induces cell death (14, 67, 71). Furthermore, this approach has been carried out by using a suicide gene which encodes for a modified human Caspase 9 (iCas9), which is strongly recognized by the bioinert synthetic molecule (B/B Homodimer, AP20187), alone or delivered by mesenchymal stromal cells (MSCs), inducing a dimer formation and activation apoptosis pathway in breast and lung cancer cells (72–74).

## Directing Gene Therapy in Cancer

In recent years, gene therapy has had considerable progress. However, the big challenge still remains: for gene therapy to be successful, the TNAs must be delivered and expressed within the target cells (75). In order to direct gene expression of TNAs on the desired cells or tissues, a specific carrier of TNAs must be developed, that is, a genetic vehicle must be designed in a fashion that it can target specific cells, such as cancer cells, and deliver TNAs in a localized manner (76–79). The second approach has to do with the presence of regulatory sequences which direct gene expression only within the target cells, even if the recombinant DNA molecule can enter cells whether they are cancer cells or not.

An important feature that determines cancer-focused gene therapy success is the regulatory sequence which controls gene expression. Because of this, expression of therapeutic genes must be controlled by cell or tissue specific promoters (80, 81). Promoters are *cis*-acting regulatory regions which direct transcription of mRNAs that, in turn, are translated into proteins. Functionally, a promoter is a DNA sequence located upstream the 5′ end of the coding region of a gene that includes the binding regions for transcription factors (82). Currently, they are being highly studied in biotechnological processes, since they cannot only increase transcriptional activity but also provide additional levels of control, *e.g.*, at the expression or stage-specific level of a gene in a particular organ or tissue. Ideally, such a promoter should provide the maximum specific expression of the therapeutic gene in the target tissue and must be strong enough to ensure the safety and efficiency of the system.

Some promoters show a specific activity only in certain cell types, making them potential candidates for transcriptional targeting. Promoters which can be used to transcriptionally target cancer can be classiﬁed into three different categories: tissue-speciﬁc, cancer-speciﬁc, and tumor-speciﬁc (76, 82).

### Tissue-Specific Promoters

Transcriptional targeting by using tissue-speciﬁc promoters resorts to promoters of genes which are specifically active in certain tissues. Although they have been widely studied for its use in cancer gene therapy (83, 84), one of the main limitations of this type of promoter is that gene expression may lead to cytotoxic effects in normal as well as in the tumor tissue derived from the same cell type. Therefore, the use of such promoters must be restricted to tissues in which damage is not critical for the survival of the host, for example, prostate, melanocytes, or thyroid. If the tissue/organ is critical, then therapeutic genes must be delivered directly to the tumor site to prevent normal tissue to be affected (85). Nonetheless, the use of cancer- and/or tumor-speciﬁc promoters rather than tissue-speciﬁc promoters could be the best option to avoid adverse effects in normal cells (86).

### Cancer-Specific Promoters

One of the main obstacles to current cancer therapies is the lack of tumor specificity. So, specifically targeting gene expression to tumor cells is one of the most important goals of cancer gene therapy (82, 85). Cancer-specific promoters are those that are functional for various types of cancers without any particular tissue/tumor specificity. However, their main feature is that they are functional within cancer cells but have no activity in normal cells. Telomerase was the first gene to be classified as cancer specific and whose promoter (hTERT) has been used to drive the expression of genes selectively in a wide variety of tumor cells (85, 87, 88). It has been observed that ~90% of human cancers express high levels of telomerase, while its activity is generally absent in normal somatic cells (89, 90). Whereby, this promoter clearly has a real potential in targeting a wide range of different tumor types.

At present, other cancer specific promoters have been studied, among them is epidermal growth factor receptor (EGFR), human epidermal growth factor receptor/neu (HER2/NEU), vascular endothelial growth factor receptor (VEGFR), folate receptor (FR), transferrin receptor (CD71), mucines, tumor resistance antigen 1-60 (TRA-1-60), cyclooxygenase (COX), cytokeratin 18, cytokeratin 19, survivin and chimeric antigen receptors (CAR) (82, 91–93). Most of the genes controlled by these promoters are over-expressed in cancer cells. That is why genetic constructions have been designed using these promoters to direct gene expression of suicide genes only in tumor cells for cancer gene therapy strategies (14).

### Tumor-Specific Promoters

**Figure 1:**
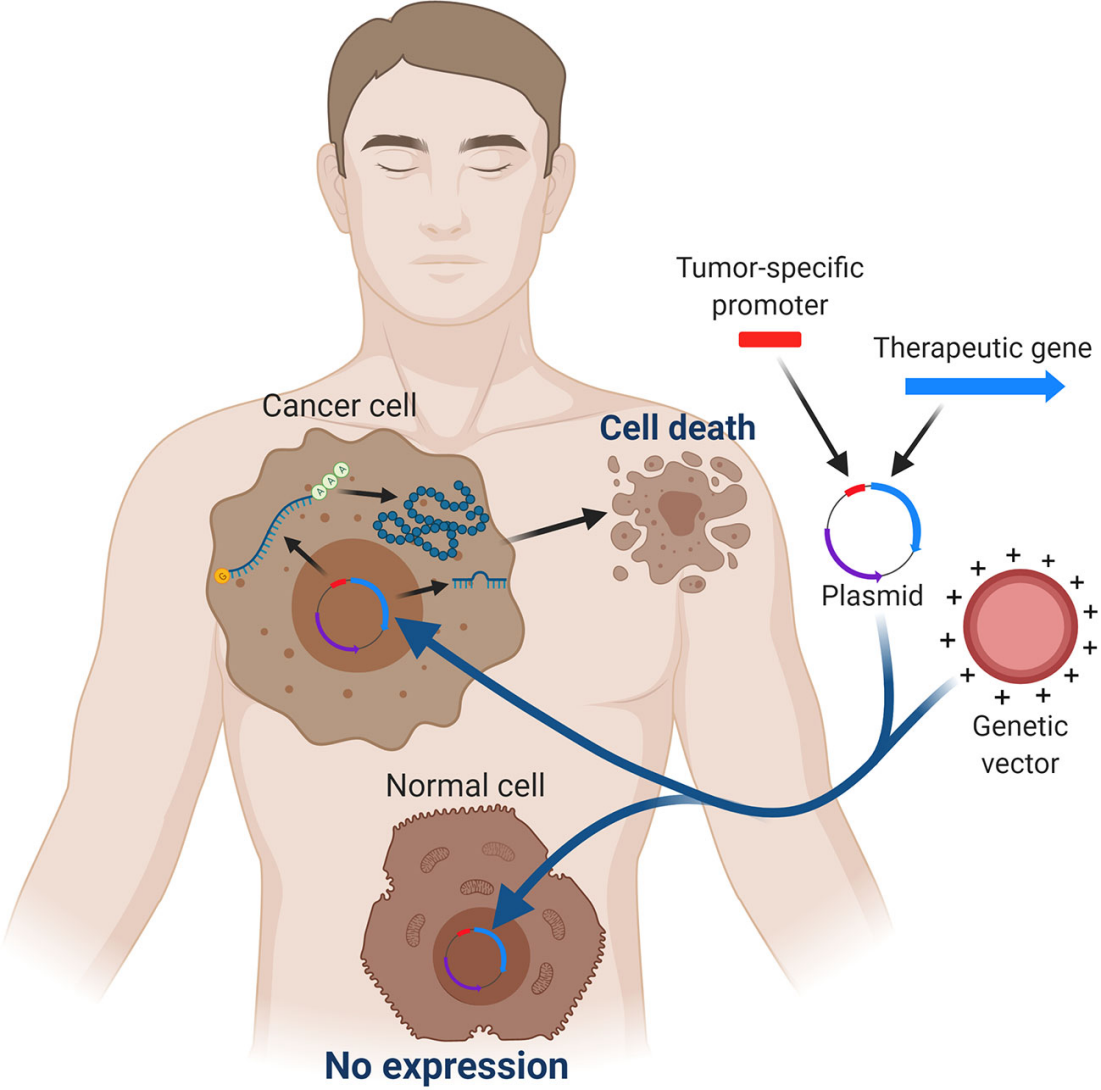
The principle of targeted gene expression controlled by a tumor-specific promoter. Specific transcription factors in cancer cells are able to induce therapeutic gene expression. On the other hand, expression of the therapeutic gene is not obtained in normal cells because they lack the specific transcription factors. Created with BioRender.com.

Tumor-specific promoters are those which are active in a limited type of cancer cells and their activity varies widely in different tumors. Nevertheless, their main feature is that they are little or non-active in normal cells (**Figure 1**) (13). So it can be ensured that tumor-specific promoters are specific for a malignant process but show no specificity for a certain type of tissue given that they respond and are activated by the tumor microenvironment. Within this group are alpha-fetoprotein promoter (AFP), thyroid transcription factor 1 (TTF-1), glypican-3 protein (GPC3), human secretory leukocyte protease inhibitor (hSLPI), ERBB2, Mucin 1 (MUC1), L-plastin, *α* lactalbumin (LALBA), cyclooxygenase 2 (COX2), epithelial glycoprotein (EPG2), A33, uPAR, carcinoembryonic antigen (CEA), breast cancer 1 (BRCA1) and BRCA2 (85, 94).

### Genetic Vectors

With the development of a wide variety of recombinant DNA technologies, as well as a better understanding of genetics and molecular biology, the promise of treating genetic diseases in order to cure them or to improve quality of life of the patients seems to be a closer reality. Nevertheless, there is still a long way to go. For gene therapy to be successful, one of the most important things to take into consideration is the use of appropriate gene delivery systems. One of those issues related with gene therapy is the need of delivering therapeutic nucleic acids into the target cells, tissues and/or organs in a safe and efficient way. To do so, it is imperative to develop strategies which allow us to specifically target the delivery of the therapeutic nucleic acids and in doing so, maximizing cell transfection. As of 2017, around 70% of all gene therapy clinical trials were carried out by viral vectors. However, there are several significant concerns regarding the application of viruses as a carrier, including immunogenicity, insertion mutagenesis, as well as reports of deaths following administration of viral vectors for gene administration (95). Therefore, considerable attention has been paid to the application of non-viral vectors with the ability to specifically direct therapeutic nucleic acids to the target cells, promoting the entry and release of these within cells, to obtain the desired biological effect (96–102).

## Most Common Cancers Treated With Gene Therapy: The Potential of Specific Promoters and Genetic Vectors

As a new promising strategy in gene therapy, the use of cancer/tumor-specific promoters for targeting TNAs has grown in recent years. Several studies have proven that, by using these types of promoters, along with different molecular strategies and delivery vectors, TNAs can be delivered safely and efficiently within the desired cells, allowing the treatment of cancer without harming healthy tissues (**Table 1**).

**Table 1:** Features of tumor-specific promoters and type of gene used in cancer gene therapy.

Cancer type	Promoter	Type of gene used	Specificity	Advantages	Disadvantages	References
**Hepatocellular carcinoma**	AFP	Increase sensitivity (NIS); Suicide gene HSV1-tk; shRNA against Beclin1	High activity in liver cancer	Tumor specificity	Weak promoter	(11, 87, 103–108)
	EA4D (enhanced AFP)	Apoptotic protein tBid	High activity in liver cancer	Stronger than wild type AFP promoter and the same tumor specificity	Useless in other types of tumors	(109)
	a2bm (variant AFP)	Oncolytic adenovirus E1A	High activity in liver cancer	Stronger than wild type AFP promoter and the same tumor specificity	Useless in other types of tumors	(11, 106, 110–112)
	hTERT	Cytotoxic gene ADI	High activity in a wide variety of tumor cells	Widely used for other tumors	Tumor non-specificity, basal expression in normal cells	(88, 112, 113)
	GPC3		High activity in liver cancer	Tumor specificity, stronger than AFP	Useless in other types of tumors	(86, 114–116)
**Breast Cancer**	ErbB2	Suicide gene HSV1-tk	High activity in breast and prostate cancer	Tumor specificity	Useful in a limited number of tumors	(117–120)
	MUC1	Suicide gene HSV1-tk	High activity in breast cancer, pancreatic cancer and cholangiocarcinoma	Higher activity in tumor cells	Basal expression in some normal cells	(121, 122)
	LALBA	Adenoviral genes E1A and E1B	High activity in breast cancer	Tumor specificity	Useless in other types of tumors	(123, 124)
**Lung cancer**	hTERT	Pro-apoptotic protein MP-VSV	High activity in a wide variety of tumor cells	Widely used for other tumors	Tumor non-specificity, basal expression in normal cells	(80)
	TTF-1	Tumor suppressor miR-7	High activity in lung cancer	Tumor specificity	Useless in other types of tumors	(125–128)
	hSLPI	Combinations: suicide gene HSV1-tk and IL-12 gene; miRNA targeting EGFR and CASP3	High activity in lung, breast and ovary cancer	Tumor specificity	Useful in a limited number of tumors	(8, 77, 129–131)
	CEA	Suicide gene E and drug PTX	High activity in lung, gastrointestinal, colorectal and breast cancers	Tumor specificity	Useful in a limited number of tumors	(13, 32, 132–134)
**Colorectal cancer**	CEA	Suicide gene E	High activity in colorectal, gastrointestinal, lung and breast cancers	Tumor specificity	Useful in a limited number of tumors	(135–138)
	COX-2	Tumor suppressor 15-PGDH	High activity in colorectal cancer	Tumor specificity	Useless in other types of tumors	(12, 91, 139–142)
	A33	Adenoviral gene E1A	High activity in colorectal, intestinal-type gastric and pancreas cancer	Higher activity in tumor cells	Tumor non-specificity, basal expression in normal cells	(138, 143, 144)
	hTERT	Adenoviral gene E1A; combination of suicide gene HSV1-tk and IL-18 gene	High activity in a wide variety of tumor cells	Widely used for other tumors	Tumor non-specificity, basal expression in normal cells	(69, 71, 145, 146)
	uPAR	Suicide gene HSV1-tk	High activity in a wide variety of tumor cells	Widely used for other tumors	Specificity in the invasive edge of a tumor	(147–150)
	FGF18	Suicide gene HSV1-tk	High activity in a wide variety of tumor cells	Widely used for other tumors	Tumor non-specificity, basal expression in normal cells	(150–152)
	KDR	Combination of suicide genes HSV1-tk and CD	High activity in a wide variety of tumor cells	Widely used for other tumors	Tumor non-specificity, basal expression in normal cells	(138, 153)
**Pancreatic Cancer**	CCKAR	Pro-apoptotic gene BikDD	High activity in pancreatic cancer	Tumor specificity	Useless in other types of tumors	(154, 155)
	MUC1	DTA toxin	High activity in pancreatic cancer, breast cancer and cholangiocarcinoma	Higher activity in tumor cells	Basal expression in some normal cells	(156–158)
	hTERT	Oncolytic adenovirus Telomelysin	High activity in a wide variety of tumor cells	Widely used for other tumors	Tumor non-specificity, basal expression in normal cells	(89, 90, 159, 160)
	SHIP1		High activity in pancreatic adenocarcinoma that overexpresses PDX-1	Stronger than wild type insulin promoters	Tumor non-specificity, basal expression in normal cells	(10, 161)
**Prostate cancer**	GRP78	Suicide gene HSV1-tk	High activity in prostate, gastric, breast, pancreatic, lung and colon cancers	Tumor specificity	Useful in a limited number of tumors	(162–164)
	hON-522E	Suicide gene HSV1-tk	High activity in prostate cancer and in a wide variety of other tumor cells	Higher activity in tumor cells	Tumor non-specificity, basal expression in normal cells	(165, 166)
	PSA	Suicide gene thymidine kinase; apoptotic protein Apoptin	High activity in prostate cancer	Higher activity in tumor cells	Tumor non-specificity, reported expression in normal cells	(167–170)
	PSMA	Apoptotic protein Apoptin	High activity in prostate cancer	Higher activity in tumor cells	Tumor non-specificity, reported expression in normal cells	(167, 168, 170)

### Hepatocellular Carcinoma

Hepatocellular carcinoma is one of the four types of cancer with the highest death rate worldwide causing 781,631 deaths per year (1); this is because of a late diagnosis and ineffective treatments as well as those which give rise to adverse effects. Target gene therapy seems to be a promising approach (171).

Cancer/tumor-specific promoters impede gene expression of the therapeutic gene within normal cells, reducing toxicity and all the same sustaining anti-cancer efficacy. AFP promoter is the most common tumor-specific promoter used in HCC gene therapy due to the high level of activity in this cancer. AFP promoter is usually active in the fetal stage and then suffers inactivation 6 months after birth. Notwithstanding, it can be reactivated in abnormal conditions like cirrhosis and certain types of cancer such as HCC or, less importantly, pancreatic cancer and lung cancer (11, 87, 103, 104, 172). For that reason, it has been widely used in gene therapy for HCC in order to direct the expression of genes such as sodium/iodide symporter (NIS) with the purpose of improving radiotherapy efficiency (105, 106) and HSV1-tk gene to increase tumor-sensitivity facing chemotherapy (103, 107).

AFP promoter has also been used in RNAi strategies (AFP-Cre/LoxP-shRNA), obtaining a specific shRNA against the cell death and autophagy regulatory protein (Beclin1) gene mRNA, inhibiting translation and HCC growth (108). Furthermore, a more transcriptionally active variant of AFP promoter (EA4D) was found and by means of the genetic construction pGL3-EA4D-tBid/H, the growth of HCC AFP+ tumors was specifically and remarkably inhibited. Nonetheless, there was no effect on AFP− tumors (109).

The main drawback of AFP promoter is that it is a weak promoter when compared to strong promoters such as CMV or CAG promoters (106, 110–112). Due to this disadvantage, its usefulness in gene therapy assays was limited. As a result, chimeric variants of AFP promoter bearing enhancers have been developed, deriving in the modified AFP promoter known as a2bm. This variant promoter bears two enhancer A and one enhancer B regions and as a result a 500-fold increase in transcription rate was obtained, besides being HCC specific. Later, AFP-a2bm was further modified using hypoxia-response elements (HRE), increasing transcriptional activity under hypoxic conditions. This allowed to overcome the hypoxic tumor environment and to target HCC with high specificity, proving it as a promising candidate for HCC treatment based on gene therapy (11).

Human telomerase reverse transcriptase (hTERT) promoter is a type of cancer-specific promoter; this can be used to drive the expression of genes in a wide variety of tumor cells without any particular tumor-specificity and has also been used to target therapeutic genes towards HCC along with arginine deaminase (ADI) gene, which encodes for an arginine-degrader enzyme (88), a potential agent against arginine-auxotroph tumors such as HCC and melanomas (113). This was done by substituting CMV promoter with hTERT promoter in an adenoviral vector aiming to direct ADI expression within the cancer cells, resulting in cytotoxicity on cancer cell lines and even eliminating tumors after two weeks of treatment in a mouse model (88). This gene therapy procedure with ADI-PEG20 (ADI PEGylated with PEG 20,000) has already passed phase I/II clinical trials but due to its low efficacy and specificity has been used only as an adjuvant therapy (112, 113).

Another tumor-specific promoter for HCC is the GPC3, an oncofetal protein belonging to the proteoglycan family only expressed in fetal development. However, the expression of this protein is reactivated mostly in HCC but also has been observed in malignant melanoma, neuroblastoma, and colon cancer (114–116). GPC3 promoter activation in HCC was demonstrated using luciferase and enhanced yellow fluorescent protein (eYFP) reporter genes in HCC cell lines and compared to normal hepatocytes (86).

Prostate and breast cancer over-expressed 1 (PBOV 1) encodes for a protein which is quite over-expressed in many types of cancer but not in normal tissues (173). Nevertheless, its role in the initiation and progression of hepatocellular carcinoma (HCC), was unknown (174). In order to reveal the role of this gene in HCC, a study was carried out in which a PBOV-1 plasmid and a PBOV-1 siRNA plasmid were delivered into HCC cells so that its expression levels and its effects on growth and metastasis could be investigated. Then, the need for an efficient and safe genetic vehicle arises. Epidermal growth factor receptor (EGFR) is a cell transmembrane protein which is known to be over-expressed in many epithelial tumors (175). So, anti-EGFR monoclonal antibodies could be potent ligands directing therapeutic nucleic acids towards epithelial tumors such as HCC. An EGFR single-chain antibody-modified graft copolymer of polyethylene glycol (PEG) and polyethylenimine (PEI) complexed with superparamagnetic iron oxide nanocrystals (SPION) was developed. The use of EGFR single-chain antibody improves tumor-targeted gene delivery, and the use of polymers is useful for nucleic acids protection against the nuclease activity *in vivo*. Nevertheless, the cationic and non-biodegradable characteristics of PEG and PEI remain to be an obstacle to overcome when it comes to its use in clinical trials (176, 177).

### Breast Cancer

Breast cancer is the result of an abnormal and disordered growth of epithelial cells of mammary ducts or lobules and is characterized by metastasis capability, being mainly a hormone-depending disease (65% of all breast cancer cases) (1, 2). Due to lack of early diagnosis and timely treatments, it is the fifth cause of cancer death worldwide. Therefore, different directed gene therapy strategies have been developed using tumor-specific promoters, achieving encouraging results in breast cancer treatment.

ERBB2 protein is an oncoprotein that belongs to the EGFR family (117). It is over-expressed in about 20% of invasive breast cancers. Particularly, it has been shown that ERBB2 over-expression boosts invasion and metastasis of breast cancer and is correlated with poor survival of patients (118). Identification of the deregulated ERBB2 pathway in breast cancer pathogenesis has led to the development of ERBB2 targeted therapies. In a study, HSV1-tk gene under ERBB2 251 bp promoter (p256-TK) transcriptional control was transfected in breast cancer cells, resulting in a higher ganciclovir sensibility without affecting normal cells (119, 120).

The MUC1 gene encodes a mucin-like high molecular weight glycoprotein and is over-expressed in breast cancer and cholangiocarcinoma (121). It has a 114 bp enhancer region capable of modulating heterologous promoter transcription. It has been shown that positive DF3 breast cancer cell lines are more susceptible to cell death by GCV when HSV1-tk is delivered and driven by this enhancer. Afterwards, a replica of the expression vector was constructed and introduced in an adenovirus vector to be delivered to breast cancer cells, inhibiting tumor growth and intraperitoneal metastasis in a breast cancer mouse model (122).

LALBA is a protein that regulates lactose production in the milk of most mammals. It constitutes the regulatory subunit of the lactose synthase heterodimer (LS), whereas *β*-1, 4-galactosyltransferase constitutes the catalytic domain. The dimer allows LS to synthesize lactose by transferring galactose residues to glucose (123). LALBA is breast specific and expresses in more than 60% of breast cancer tissues. LALBA promoter showed a significantly higher activity in MDA-MB-435S and T47D breast cancer cell lines when compared against normal breast cell lines or other tumor cell lines. Furthermore, the replication efficiency of the vector and as a consequence its tumor cell destroying capability were increased as shown versus normal cell lines (negative LALBA promoter cells) (124).

Cationic porphyrin microbubbles (CpMBs) have been synthesized from a porphyrin grafted lipid which has two cationic amino groups (PGL-NH2) and the fluorocarbon inert gas C3F8. This design has two purposes: first of all, the porphyrin group can be used as a photosensitizer in order to carry on photodynamic therapy (PDT); secondly, the amino groups provide positive charges which can interact with a siRNA that can be used for FOXA1 knockdown (FOXA1 KD) in estrogen receptor positive breast cancer cells (ER + BC cells) (178, 179). *In vivo* experiments were carried on in which female Balb/c nude mice were injected with cells from the MCF7 cancer cell line and then subjected to treatment with CpMBs/siRNA followed by ultrasound targeted microbubble destruction (UTMD) being guided by contrast enhanced ultrasound (CEUS) (180). Promising results were obtained with this novel CpMBs in combination with ultrasound technology, which lead to a more efficient accumulation of porphyrin and siRNA into tumor cells (181, 182).

New approaches aim to treat breast cancer using co-delivery systems which can transport drugs and therapeutic genes within breast cancer cells. Among the many gene delivery systems developed, inorganic materials are highly promising. Hydroxyapatite nanoparticles have shown many advantages such as low cytotoxicity, wider surface areas and they are easy to fabricate and modify (183). Amine-functionalized hydroxyapatite nanoparticles (NHAP) were synthesized from 3-aminopropyl-triethoxysilane (APS) and HAP nanoparticles. Then, candesartan (CD) and p53 plasmid were added to give a drug–gene co-delivery vehicle. These nanoparticles showed the desirable characteristics of surface charge and particle size good enough to provide pDNA condensation and protection. After 72 h post-incubation in *in vitro* assays, cells treated with these nanoparticles showed viability above 90%. Transfection efficiency of these nanoparticles was about 26%. Finally, the design of this co-delivery system showed a strong inhibitory effect on angiogenesis *in vitro*, and *in vivo* analysis demonstrated a superior anti-tumor effect in a mouse model (181, 184, 185).

### Lung Cancer

Lung cancer is the main cause of cancer deaths worldwide, accounting for 1,761,007 deaths from the 2,093,876 new reported cases in 2018 (1) despite advances in chemotherapy, surgery, and radiotherapy.

The matrix protein (MP) of the vesicular stomatitis virus (VSV) induces apoptosis in tumor cells in the absence of other viral components. Wild-MP gene was used to construct pVAX-M recombinant plasmid, which showed an efficient suppression of malignant tumors growth by inducing apoptosis in *in vivo* and *in vitro* assays. Afterwards, phTERTM plasmid encoding VSV MP under transcriptional control of hTERT promoter was constructed, displaying the same anti-tumor effect but specifically directed against lung adenocarcinoma (80).

On the other hand, thyroid transcription factor 1 (TTF-1) is a member of the Nkx2 transcription factors family, classified as a tissue-specific oncogene given that it is expressed mainly in lung cancer cells but not in other types of cancer and whose expression levels are tightly linked with patient prognosis (125). Based on the above, a miR-7 expression vector under TTF-1 promoter transcriptional control was constructed (p-T-miR-7), this expression vector displayed a reduction of tumor growth rate, migration and metastasis of lung cancer cells *in vivo* and *in vitro* suggesting the usefulness of miR-7 to develop new gene therapy strategies selectively against lung cancer (126–128).

As discussed previously, suicide gene therapy is another interesting approach in cancer gene therapy. From this point of view, an expression vector was constructed from HSV1-tk and human interleukin-12 genes under transcriptional regulation of tumor-specific hSLPI (human secretory leukocyte protease inhibitor) promoter, which is known to be active in lung, breast, and ovary cancers (8, 77, 129). This vector displayed a more specific anti-tumor effect because of hSLPI promoter transcriptional regulation, besides demonstrating that suicide gene therapy combined with immune gene therapy provides a stronger anti-tumor effect than gene therapy using a single gene (77). Otherwise, a recombinant adenovirus (Ad-SLPI-EGFRamiR-SLPI-revCASP3) expressing an artificial miRNA targeting EGFR and recombinant caspase-3 (CASP3) under transcriptional regulation from SLPI promoter was constructed. This displayed a specific novel anti-cancer gene therapy strategy which combines EGFR inhibition as well as CASP3 induced apoptosis (130, 131). The inhibitory effect caused by this adenovirus was commensurable to the therapeutic effects of cis-platinum and cetuximab (8).

CEA belongs to a cell-surface glycoproteins family and is the most used tumor marker in clinical diagnosis of colorectal, gastrointestinal, lung and breast cancers (32). It is normally expressed in epithelial cells of the fetal gastrointestinal tract (132, 133); however, non-small-cell lung carcinoma (NSCLC) patients have elevated CEA serum levels, something that has been correlated with low survival rates (13, 134) so that CEA promoter has been used in directing E gene (pCEA-E) along with (PTX) in order to specifically target lung cancer cells, improving anti-tumor effects of PTX. *In vivo* assays corroborated this combined therapy effectiveness and demonstrated that CEA is an excellent tumor-specific promoter for targeting therapeutic genes expression within lung cancer cells inducing apoptosis and with no harm to normal cells (13).

Human Wnt inhibitory factor-1 (hWIF-1) has been described as an effective anti-oncogene useful for NSCLC gene therapy. The use of viral vectors to deliver these therapeutic genes into NSCLC cells has failed, given the inherent disadvantages of these genetic vehicles such as immunogenicity and insertional mutagenesis. So, a novel genetic vehicle based on PEI and branched PEI1800 coated with SP5-2 peptide, which specifically targets NSCLC cells, was developed. When proved on A549 cells, this vehicle provided a 50% transfection efficiency, showing, this way, that this is a promising genetic vehicle which can be useful for delivery of therapeutic nucleic acids on cancer cells (98).

Nanocarriers, such as nanostructured lipid carriers (NLC), have proven to be potential candidates to work as non-viral genetic vehicles due to important features such as increased chemical stability, higher loading capacity of nucleic acids, lower cytotoxicity and controlled release (186). Another strategy used in the design of safer and more efficient genetic vehicles is the construction of dual ligand-decorated lipid carriers. Transferrin (Tf) is a protein which has been used for targeted gene therapy, given that most cancer cells of lung carcinoma overexpress transferrin receptors (187). In a similar way, hyaluronic acid (HA) has also been used for a similar purpose for most of the non-small cell lung cancer (NSCLC). Then, for the sake of finding a more promising genetic vehicle, transferrin and hyaluronic acid containing polyethylene glycol-distearoyl phosphoethanolamine (PEG-DSPE) ligands were synthesized. The systemic delivery efficiency of nucleic acids using this novel genetic vehicle was evaluated *in vivo* in a human lung adenocarcinoma A549 cell-bearing mouse model. These nanocarriers showed a sustained release of pDNA, which can lead to the persistence of the therapeutic effect when used for *in vivo* purposes. Even more, the presence of the Tf and HA ligands on the surface of NLC granted them lower cytotoxicity when compared with uncoated NLC (100, 101).

### Colorectal Cancer

Colorectal cancer (CRC) originates in colon and rectum, usually starting with the forming of a polyp because of an epithelial proliferation from colon and rectal mucosa. The probability for a polyp of turning into a malignant neoplasia depends on the type of polyp according to its histology. Adenomatous polyps are likely to turn into cancerous neoplasia because of their pre-cancer nature for being a type of adenoma. Meanwhile, inflammatory, and hyperplasic polyps are not considered as pre-malignant lesions (188). CRC is the third type of cancer with the highest incidence worldwide and the second one on the list of cancers with higher death rate (1, 2).

As in lung cancer, CEA is an oncofetal tumor-marker over-expressed in more than 90% of CRC cells. High CEA levels have been found in serum as well as high levels of its mRNA in end-stage CRC patients (135). CEA levels have been used in predicting and keeping track of recurrence and metastasis of CRC in stage-II patients (136, 137). CEA promoter has been used to direct therapeutic gene expression in CRC cells, such as E gene against colon cancer, triggering a high inhibition of cell growth compared to normal human colon cells. Moreover, it has been shown in mice carrying subcutaneous MC-38 colon cancer cells that there is a significant decrease in tumor size and low Ki-67 levels compared against untreated tumors (138).

COX-2 is an enzyme that catalyzes initial oxidation of arachidonic acid for prostaglandins synthesis, an essential factor in carcinogenesis and tumor evolution. COX-2 is over-expressed in 93% of colon cancers and in 87% of rectum cancers (139). Over-expression levels of COX-2 have been shown to be related to cancer progression and death rate in patients with CRC (140, 141). COX-2 gene promoter has been found to be active in CRC cell lines but not in normal human intestinal epithelial cell lines, via analysis of its transcriptional activity using luciferase reporter gene (91). This promoter has also been used to control 15-hydroxyprostaglandin dehydrogenase (15-PGDH) gene expression, a repressed gene in most cancers. By doing so in colon cancer cells, growth and migration of CRC cells was inhibited (12, 142).

A33 protein is a member of the transmembrane protein family of the immunoglobulin superfamily, only found in the small intestine and colon, associated with gut immune response, cell adhesion processes and cell traffic. A33 protein over-expression is correlated with many cancers such as primary and metastatic CRC (95%), diffuse gastric cancers (63%), intestinal-type gastric cancers (83%) and pancreas cancer (50%), nonetheless it has not been found in normal colon epithelium (138, 143). The A33 promoter has been used to specifically drive the expression of the E1A anti-cancer protein gene to decrease tumorigenic potential, inhibit cell growth and activate apoptosis in cancer cells. Production of favorable levels of E1A mRNA has been demonstrated in different CRC cell lines, but not in normal colon cells, with a slight activity in HCC and melanoma cell lines, so that the A33 promoter can be used as tumor-specific promoter (144).

The hTERT is synthesized in cells with high levels of enzymatic activity (*e.g.* tumor cells) but not in normal tissues (145). Telomerase is highly active in malignant tumors and high levels of hTERT mRNA have been correlated with poor prognosis for CRC patients versus patients with low telomerase levels (146). hTERT promoter has been used in directing therapeutic gene expression such as E1A, showing a high specificity towards CRC, inhibiting 75% of cancer cell growth, obtaining apoptosis and necrosis levels of 32.3 and 31.5% respectively (71). Furthermore, combined gene therapy with interleukin 18 (IL-18) gene and HSV1-tk under hTERT promoter transcriptional control has been carried out, this strategy confers a specific anti-tumor immunity, partially or completely eliminating tumors (69).

The uPAR gene encodes for a serine protease that catalyzes inert zymogenic plasminogen into plasmin. It has been observed that the uPAR gene is positively regulated by activated RAS signaling pathway, the main signaling pathway in CRC (147). This gene is over-expressed in many tumors such as those of pancreas, liver, breast and especially gastrointestinal (148). The specific tumor union of activator protein (AP-1) to uPAR promoter has been found in approximately 40% of CRC patients and 38.9% of them showed this specific tumor union in resected tumors in contrast to low or absent attachment in the corresponding normal mucosa, demonstrating the specific tumor activity of uPAR in CRC and not in normal tissues (149). uPAR promoter specific activity has been demonstrated in colon cancer cell lines (SW480) and CRC (HTC116) by means of lacZ reporter gene. By administering HSV1-tk under uPAR promoter transcriptional regulation within SW480 and HCT116 cell lines cell growth rate decreased significantly by ganciclovir administration (150).

Fibroblast growth factor 18 (FGF18) is a crucial mitogen in the embryonic stage taking part in bone and cartilage development (151). Its over-expression has been linked with different types of cancer, mainly in CRC, promoting the transition of colon carcinogenesis from adenoma to carcinoma (151, 152). Activity of FGF18 promoter has been tested with *lacZ* reporter gene in SW480 and HCT116 cell lines versus normal human umbilical cord colon cells, and it was found that galactosidase activity was much higher in cancer cells than in normal cells. Moreover, specific tumor activity of FGF18 promoter was demonstrated by expressing HSV1-tk gene within CRC cells, significatively inhibiting its growth after treatment with ganciclovir (150).

The receptor that contains the endothelial cell type specific tyrosine kinase domain (KDR) is the receptor of the vascular endothelial growth factor (VEGF), which plays a vital role in the growth and development of endothelial cells. KDR expression has been detected in a wide variety of cancer cells and vascular endothelial cells but not in normal cells (153). By screening the expression of CD and HSV1-tk genes (KDR/CD-TK) in colon cancer cells, KDR promoter expression has been proven to be specific for this type of cells, finding high levels of CD/TK mRNA in SW480 and SW620 (KDR positive human colon adenocarcinoma) which were found to be highly susceptible to 5-FC and ganciclovir prodrugs and with no effect on LS174T cells (KDR negative human colon adenocarcinoma) (138).

Copolymers are becoming more attractive to scientists due to the features and advantages that emerge when using new combinations of monomers. In colorectal cancer gene therapy, a novel nanocarrier was developed using a copolymer of poly-(ethylene glycol)--poly-(caprolactone) which were used to co-loading 5-Fluorouracil and the enhanced green fluorescent protein coding gene. Transfection efficiency was tested on colorectal cancer-bearing mice, showing 70–90% of transfection percentage 24 to 72 h post-transfection, respectively (99, 189).

An interesting approach for colorectal cancer (CRC) gene therapy and, specifically, in the development of novel genetic vehicles is the construction of nanoparticles (NPs) made of mesoporous silica nanoparticles (MSNs) which can be modified using polymerized dopamine and AS1411aptamer (190). The use of the AS1411aptamer confers these nanoparticles high tumor specificity, given that this aptamer binds specifically to nucleolin, which is a protein over-expressed on the cell surface of many types of tumor, including CRC (191). *In vivo* and *in vitro* assays demonstrated that these NPs can effectively target CRC cells (98).

### Pancreas Cancer

Pancreas cancer is one of the most aggressive malignant human neoplasia and the seventh cause of death due to cancer worldwide accounting for 458,918 of new cases and 432,242 deaths in 2018 (1), given the lack of proper therapies (82).

Cholecystokinin type A receptor (CCKAR) promoter has relatively high activity in pancreatic cancer cells when compared with normal cells, this tumor-specific promoter was modified for enhancing its activity to be used within pancreas cancer cells for directing BikDD expression, a powerful pro-apoptotic gene, demonstrating its effective and specific anticancer effect (154). Furthermore, the versatile expression vector “VISA” (VP16-GAL4-WPRE integrated systemic amplifier) which contained the same tumor-specific CCKAR promoter was constructed to direct the expression of BikDD in pancreas cancer *in vivo*. The targeted expression of BikDD by the CCKAR-VISA vector showed a significant antitumor effect in pancreatic cancer and prolonged survival in the mouse model used (155).

A strategy with Diphtheria toxin A (DTA) against pancreatic cancer has also been developed, using the tumor-specific promoter MUC1, due to its over-expression in pancreas ductal adenocarcinoma (PDA) and its association with tumor aggressiveness (156). This strategy has been used to direct the expression of DTA only within tumor cells, since this toxin inhibits protein synthesis and is lethal for cells (157). However, some normal cell types, such as gastrointestinal and breast epithelial cells, express MUC1 (158), so this pMUC1/DTA construct may cause gastrointestinal side effects in treated patients.

Telomelysin (OBP-401) is an oncolytic modified adenovirus in which the hTERT promoter controls viral replication, therefore it only replicates within cells that overexpress hTERT such as pancreatic cancer cells (89, 90, 159). Telomelysin has been shown to effectively lysate pancreas cancer cells and reduce xenograft tumors in murine models by itself as well as in combination with docetaxel (160).

With the purpose of developing a more efficient pancreatic cancer gene therapy, a synthetic human insulin super-promoter (SHIP1) was designed to improve the activity and specificity of the human insulin promoter. SHIP1 has been shown to be a promoter with higher activity than that of endogenous human insulin promoters and rat insulin promoters (RIP), which are used to direct expression in pancreatic adenocarcinoma that overexpresses pancreas and duodenal Homeobox gene 1 (PDX-1) (10, 161). This new gene therapy strategy using synthetic super-promoters could be used to more efficiently direct therapeutic genes expression in various types of cancers.

An interesting approach in the development of genetic vehicles for treatment of cancer is the use of exosomes to enhance targeting of oncogenic Kras in pancreatic ductal adenocarcinoma (PDAC). Exosomes, which are vesicles of nanometric dimensions and are produced by all cells, were obtained from cell cultures of human foreskin fibroblasts (BJ) (192). Then, these exosomes were electroporated with a siRNA or a plasmid for shRNA silencing of KRAS mutations, which are the key driver of pancreatic cancer (193). After that, their biological effect was evaluated in *in vitro* and *in vivo* essays. Exosomes are characterized by the presence of transmembrane and membrane anchored proteins, one of these is CD47, a protein that signals for the inhibition of phagocytosis via the interaction with the ligand for signal regulatory protein alpha (SIRPα), allowing evasion of phagocytosis by circulating monocytes and increasing half-life of exosomes in circulation. Kras mutant cancer cells are known to have an enhanced micropinocytosis activity, so that exosomes uptake is favored, allowing the targeted delivery of the therapeutic nucleic acid (96, 194–196).

One of the most recent approaches to treat cancer is the use of engineered cells by a non-viral vector which carries a therapeutic protein which induces apoptosis in cancer cells. In this new approach, human mesenchymal stem cells (hMSCs) are genetically modified with complexes of branched polyethyleneimine (bPEI) and TRAIL (tumor necrosis factor-related apoptosis-inducing ligand) gene (197). To overcome the low transfection efficiency of the TRAIL-gene vector within hMSCs, photochemical internalization (PCI) was the method utilized to carry on genetic modification. After genetically modifying hMSCs, the transfection and secretion of TRAIL protein into culture supernatants was evaluated as well as the evaluation of *in vivo* therapeutic effect in tumor-bearing mice and histologic analysis. TRAIL is a member of the tumor necrosis factor (TNF) superfamily which is able to form homotrimer with death receptors (DRs) on the cell membrane; when it does, it triggers apoptosis pathway in cancer cells and has a negligible effect on normal cells (198). To overcome the limitations of *in vivo* application of DNA complexes with polymers, hMSCs were applied for direct secretion of TRAIL protein; these cells were used given their ability for homing to tumor sites and their immunity privileges which prevent them from being rejected *in vivo*. Finally, the use of PCI is justified because it maximizes cellular internalization of DNA-bPEI complexes; this technique uses near infrared light (NIR) along with a photosensitizer, which enhances the cell membrane permeability and allows photo-induced endosomal escape efficiency for enhanced gene transfection (97, 199–201).

### Prostate Cancer

Prostate cancer is the second type of cancer with the highest incidence and the fifth cause of death among men worldwide, taking 358,989 lives per year (1) and representing a high risk for elder men.

One promoter used in the development of gene therapies against tumor cells of prostate cancer is the glucose-regulated protein 78 (GRP78) promoter. This promoter is inactive in healthy adult tissues but it has been proven to be highly active in a wide range of cancer cells like prostate, gastric, breast, pancreatic, lung and colon (162–164). One strategy developed consisted in using GRP78 promoter as the regulatory sequence in the HSV1-TK suicide gene. Under the enzymatic action of the protein encoded by this gene, GCV is transformed into GCV-monophosphate and then into GCV-diphosphate and GCV-triphosphate. This last product showed cytotoxic effects specifically on prostate cancer cells (164).

Another promoter receiving attention is the human osteonectin promoter (hON-522E). This promoter regulates transcription of osteonectin, a protein known to play roles in cell adhesion, proliferation and migration. Osteonectin is over-expressed in many types of cancer such as prostate cancer, where it is involved in metastasis (165, 166). A vector was constructed with hON-522E promoter regulating transcription of a HSV1-TK suicide gene, demonstrating induction of cell death *in vitro* (PC3M) and slowing the growth of prostate tumors in an xenograft model, without other organ toxicity (166).

Prostate-specific antigen (PSA) is a cytoplasmic protein present in prostate gland cells and prostatic duct epithelial cells. Its expression has been documented in normal prostate tissues but is known to be over-expressed in prostate cancer cells. On the other hand, prostate-specific membrane antigen (PSMA) is an intrinsic protein on membranes of prostatic epithelial cells and has been found to be over-expressed in prostate cancer, especially in metastasis (167, 168). Therefore, promoters of these two proteins seem to be suitable candidates for directing gene therapy in prostate cancer. One approach was constructing a plasmid containing the thymidine kinase suicide gene under transcriptional control of the human PSA enhancer/promoter fragment. Further directionality was achieved by using JC polyomavirus virus-like particles, which show tropism towards androgen receptor positive prostate cancer cells, as the genetic vehicle carrying the recombinant plasmid. The constructed plasmid could kill 22Rv1prostate cancer cells *in vitro* by inhibiting growth of these cells in a xenograft mouse model (169). In a similar way, a recombinant plasmid was constructed using PSA and PSMA regulatory elements ruling transcription of apoptin. When transfecting human prostatic adenocarcinoma cell line LNCaP, viability was significantly decreased (170).

Cationic polymers are known to interact with the negatively charged therapeutic nucleic acids, forming stable nanocomplexes. One of the most used cationic polymers is polyethylenimine, given that it binds DNA with high efficiency and has a proton sponge effect which is useful in endosomal escape of nucleic acids. Besides, it is a polymer that can be modified with targeting agents. Chlorotoxin is a peptide which binds in a specific fashion with matrix metalloproteinase-2 (MMP-2), which is over-expressed in certain types of cancer such as brain, prostate, skin, sarcoma, among others and plays a role in cancer metastasis (202). So, a novel genetic vehicle was constructed by conjugating chlorotoxin with PEI and forming nanocomplexes using a plasmid which contains a gene which encodes for melittin, a peptide present in bee venom and that has some anti-cancer activity. The transfection experiments showed that this genetic vehicle can reach transfection efficiencies of 49% with no cytotoxic effect (203–206).

Sometimes, it is necessary to use more than one genetic delivery strategy in order to archive the requirements of a safe and efficient transfection of mammalian cells. In an attempt to fulfill these needs, a complex consisting of 11-mercaptoundecanoic acid modified nanocages (AuNCs)/polyethylenimine (PEI)/miRNA/hyaluronic acid (HA), abbreviated as AuNCs/PEI/miRNA/HA was designed and constructed. Due to the presence of HA, these complexes can be specifically targeted for intracellular delivery of miRNA via HA receptor mediated endocytosis, using a layer-by-layer method. The use of HA as a mediator of endocytosis has additional advantages such as its unimportant nonspecific interactions with serum components, improving its *in vivo* availability. Attachment of PEI onto AuNPs surface making use of 11-mercaptoundecanoic acid provides the surface for the interaction with the negatively charged miRNA, besides PEI property of strong endosomal escape. Finally, the use of AuNPs has proven to be quite dynamic due to their physicochemical properties as well as the ease with which AuNPs surface can be functionally modified. Ultimately, the use of photothermal therapy, which makes use of NIR light, enhances the antitumor effect of these new genetic vehicles (98, 102, 207).

## Perspectives

As recombinant DNA technologies have arisen, the need of finding new treatments for diseases has become an important topic within the scientific community. Cancer is one of the most aggressive and deadliest diseases among humans and given its genetic basis and the lack of effective conventional treatments, it is a suitable candidate for using gene therapy. As we have reviewed in this work, there are different genetic engineering techniques which can be used for controlling or restoring normal gene expression within cancer cells. Nevertheless, that approach requires further research to be delivered safely and efficiently within cancer cells as well as being expressed just inside the tumor cells of interest rather than in normal tissues. Cancer and tumor-specific promoters have been shown to be highly effective when it comes to targeted gene expression, mainly because they direct gene expression only within a specific type of tumor cells or cancer cells and not within normal tissues, which results in targeted therapies involving cell death just for malignant tissues while keeping normal tissues safe and intact. Along with these cancer and tumor-specific promoters, the right delivery system, that is, a safe, efficient, and specific genetic vector, must be available in order to render a highly promising therapy, with specificity and efficacy enough to ensure cancer elimination and normal tissue preservation.

So, it is of pivotal importance to continue in the search of new promoters which can direct gene expression only within the desired cells and new genetic vectors that can deliver recombinant DNA molecules also in a specific fashion with no cytotoxicity and high transfection efficiencies so that gene expression is assured to take part only where needed. To do so, cancer genetics must be further studied so that the principles controlling gene expression in cancer can be fully understood and manipulated in a beneficial way via gene therapy. Simultaneously new genetic vectors must be developed and, in doing so, new materials must be studied in order to fulfil the needs of biocompatibility, no cytotoxicity, high transfection rates, tissue specificity and high loading capacity of genetic material, with the purpose of providing a new tool in cancer treatment that can (surpass the shortcomings of the conventional therapies) complement or reduce toxicity of conventional treatments available nowadays, increasing survival rates and improving life quality of patients suffering cancer.

## Conclusions

Cancer and tumor-specific promoters have proven to be an important feature in the construction of recombinant DNA molecules for cancer gene therapy. Yet, a more advanced knowledge of cancer genetics is required in order to find effective and safe elements to control gene expression. New molecules and materials for the development of genetic vectors have opened the possibilities to deliver nucleic acids into cells, nevertheless, the challenge of finding a genetic vector that is safe and efficient remains.

## Author Contributions

MM-S: Development of most topics, figure creation, and table creation. DB-E: Participation in the development of the entire gene therapy topic of the most common cancers. OM-G: Development of the perspectives chapter and participation in the drafting of the entire article. EA-H: Participation in the organization and review of the topics. MI-H: General idea, coordination and advice of the whole article. All authors contributed to the article and approved the submitted version.

## Funding

Funding obtained from COFAA-IPN, linked to SIP project #20200448: Evaluación de la transfección con liposomas que contienen un nuevo lípido catiónico y determinación de la direccionalidad de la expresión genética.

## Conflict of Interest

The authors declare that the research was conducted in the absence of any commercial or financial relationships that could be construed as a potential conflict of interest.
